# Should policy-makers and managers trust PSI? An empirical validation study of five patient safety indicators in a national health service

**DOI:** 10.1186/1471-2288-12-19

**Published:** 2012-02-27

**Authors:** Enrique Bernal-Delgado, Sandra García-Armesto, Natalia Martínez-Lizaga, Begoña Abadía-Taira, Joaquín Beltrán-Peribañez, Salvador Peiró

**Affiliations:** 1 Instituto Aragonés de Ciencias de la Salud, Instituto de Investigación Sanitaria Aragón (IIS Aragón), Zaragoza, Spain; 2 Departamento de Salud, Gobierno de Aragón, Zaragoza, Spain; 3 Centro Superior de Investigación en Salud Pública (CSISP), Valencia, Spain

## Abstract

**Background:**

Patient Safety Indicators (PSI) are being modestly used in Spain, somewhat due to concerns on their empirical properties. This paper provides evidence by answering three questions: a) Are PSI differences across hospitals systematic -rather than random?; b) Do PSI measure differences among hospital-providers -as opposed to differences among patients?; and, c) Are measurements able to detect hospitals with a higher than "expected" number of cases?

**Methods:**

An empirical validation study on administrative data was carried out. All 2005 and 2006 publicly-funded hospital discharges were used to retrieve eligible cases of five PSI: Death in low-mortality DRGs (MLM); decubitus ulcer (DU); postoperative pulmonary embolism or deep-vein thrombosis (PE-DVT); catheter-related infections (CRI), and postoperative sepsis (PS). Empirical Bayes statistic (EB) was used to estimate whether the variation was systematic; logistic-multilevel modelling determined what proportion of the variation was explained by the hospital; and, shrunken residuals, as provided by multilevel modelling, were plotted to flag hospitals performing worse than expected.

**Results:**

Variation across hospitals was observed to be systematic in all indicators, with EB values ranging from 0.19 (CI95%:0.12 to 0.28) in PE-DVT to 0.34 (CI95%:0.25 to 0.45) in DU. A significant proportion of the variance was explained by the hospital, once patient case-mix was adjusted: from a 6% in MLM (CI95%:3% to 11%) to a 24% (CI95%:20% to 30%) in CRI. All PSI were able to flag hospitals with rates over the expected, although this capacity decreased when the largest hospitals were analysed.

**Conclusion:**

Five PSI showed reasonable empirical properties to screen healthcare performance in Spanish hospitals, particularly in the largest ones.

## Background

The Spanish National Health Service, like others, has become influenced by the Patient Safety movement. Evidence from two reports on Spanish hospitals, following other international works on adverse events [1-7], inspired the debate. The first one, showed an in-patient incidence of adverse events ranging from 5.6% to 16.1%, being avoidable between 17% and 41% of them [8]. The second one, found an incidence of adverse events amenable to health care up to 10.1% [9]. As a matter of fact, these findings contributed to steer the inclusion of Patient Safety Indicators (PSI) within the sets of National and Regional Quality Indicators, being modestly used by health care authorities to assess health care performance.

The Spanish National Health Service (NHS) experience is built on the insight from the Healthcare Cost and Utilization Project by the US Agency for Healthcare Research and Quality [10] and the requirements by the OECD [11]. In spite of the efforts made in building a valid tool concerns remain about whether PSI are appropriate to inform hospital performance. Beyond the need of local adaptation [12,13], most of the caveats have pointed out to flaws in their capacity to attribute excess-cases to hospitals by detecting true incident adverse events [14-23]. Less has been written on their empirical properties, mainly because of their local nature; in particular, to what extent PSI show systematic variation on adjusted-incidence (as opposed to random) and, their ability to provide precise estimates and therefore, being sensitive to detect providers over the expected. In this sense, several works on similar topics, have partially addressed some of these issues [24-26].

This paper aims at testing the empirical properties of five PSI as well as their ability to respond relevant questions for concerned users; thus: a) Are differences in PSI rates across hospitals systematic?; b) Do PSI measure differences among hospital-providers as opposed to differences among patients?; and, c) Are measurements precise enough and able to detect providers with a higher (lower) than expected number of cases?

## Methods

### Study design, population and setting

An empirical validation study, based on administrative data, was carried out. All 2005 and 2006 publicly-funded hospital discharges were used to retrieve eligible patients. In order to reduce random noise on estimates, hospitals with less than 30 eligible cases were excluded.

Five PSI were analyzed for the purposes of this study: Death in low-mortality DRGs (MLM); decubitus ulcer (DU); postoperative pulmonary embolism or deep vein thrombosis (PE-DVT); infections due to medical care, including catheter-related infections (CRI) and postoperative sepsis (PS). The number of cases (numerators) and eligible admissions (denominators) are shown in Table 1. The election of these five indicators was based on a previous report on the validity of ARQH PSI indicators for the Spanish case [23].

**Table 1:** PSI adjusted-incidence and variation across hospitals

	Mortality in Low- mortality DRGs		Decubitus ulcer		Catheter-related infection		Postoperative PE or DVT		Postoperative sepsis	
**Cases**	683		18,738		5,375		9,727		10,602	

**Patients at risk**	1,255,647		2,190,633		2,954,018		1,949,434		612,590	

Adjusted-incidence^* ^(range P_5_P_95_)	0.54	0 to 1.41	7.69	6.00 to 12.63	1.82	1.59 to 2.20	4.99	4.20 to 6.10	17.3	15.30 to 20.26

**Statistics of variation**										

**RV_95-5_**(CI95%)	12.88	9.36-14.98	2.03	1.79-2.32	1.37	1.31-1.44	1.39	1.33-1.58	1.31	1.24-1.35

**RV_75-25_**(CI95%)	7.06	4.77-7.88	1.40	1.33-1.44	1.15	1.11-1.17	1.15	1.13-1.18	1.12	1.10-1.14

**EB **(CI95%)	0.32	0.19-0.51	0.34	0.25-0.45	1.14.	0.85-1.51	0.19	0.12-0.28	0.30	0.19-0.45

* Adjusted by: age, gender and Elixhauser's comorbidities except in PSI#2 where crude incidence is shown. Empirical Bayes statistic is a measure of systematic variation -variation beyond chance. A value different to 0 would represent systematic variation; as for its magnitude, the higher the value the more the systematic variation

For the purpose of this study a Spanish version from the AHRQ PSI algorithms was used. PSI definitions by AHRQ -4.1 version- were subject to a local validation process, accounting for differences with respect to the US healthcare system (i.e., ICD 9^th ^version and DRG version in use, as well as some coding characteristics) with a view of improving face validity for the Spanish context. Although it has been described elsewhere [23,27], it might be useful to highlight that a dedicated *consultation group *involving clinicians and coders set about to examine and adapt as needed, both numerators and denominators for each indicator. In the particular case of MLM -an empirically built indicator- the list of low mortality DRGs was re-defined for the Spanish case. The overall correlation between original AHRQ and Spanish PSI definitions as to flag events in the hospitals under study was high across the five indicators, ranging from 0.75 in PE-DVT to 0.95 in PS.

### Main endpoints

Three main endpoints were studied: a) Systematic variation defined as an Empirical Bayes value different to zero; b) Cluster effect defined as a rho statistic value different to zero; c) Sensitivity as the statistically significant difference between the observed and the expected, as provided by the residual analyses in a multilevel approach.

### Analyses

Adjusted-incidence (I) for each PSI -except MLM- and hospital were calculated. Crude incidence was used in the case of MLM due to its quasi-sentinel event nature. Variation in incidence was calculated using the ratio of variation between hospitals in percentile 95 and percentile 5 (RV_95-5_), and the ratio of variation between hospitals in percentile 75 and percentile 25 (RV_75-25_).

Methods intended to respond the aforementioned questions on PSI empirical properties were carried out, once variation in the incidence of adverse events was calculated. Hereinafter, we describe these methods.

#### Are differences across hospitals systematic rather than random?

We used an observed to expected approach, being the observed the counts of adverse events in each hospital under study, and the expected the predicted cases from a logistic regression considering as covariates the recorded age, sex and comorbidities for each patient. (An adaptation from the ARQH version [28] was used to retrieve comorbidities)

Given both observed and expected counts, the Empirical Bayes statistic (EB) was estimated following a two-step hierarchical model. The first step assumes that, conditional on the risk r_i_, the number of counts y_i _follows a Poisson distribution, y_i_|r_i _~ Poisson (e_i_r_i_), whereas in the second one, heterogeneity in rates is modelled adopting a common distribution π for the risk r_i _(or for its logarithm), r_i _~ π (r|θ), with θ the vector of parameters of the density function. EB statistic is based on the assumption that the log-relative risks are normally and identically distributed, log (r_i_) ~ N(μ, σ2).

In order to assess the alternative hypothesis, confidence intervals for the observed statistics were derived. In order to avoid parametric assumptions on the distribution of observed cases, we used a non-parametric methodology -a sampling with 2,000-time re-sampling method for each one of the simulated samples. Credibility intervals from percentiles 2.5 and 97.5 were obtained [29].

#### Do PSI measure differences among hospital-providers as opposed to differences among patients?

Classically, risk adjustment has been used to compare providers, assuming that all patients have a homogenous propensity to have the outcome of interest, wherever the place they are treated. We could otherwise hypothesize that this propensity is more similar among patients within a hospital than among patients from different hospitals -this would be the so called cluster effect. If true, classical methods ignore this effect and mislead the true estimates of variation. Alternatively, the multilevel approach considers the cluster effect (heterogeneity across hospitals) in the variance estimation, producing sounder estimates and a better understanding on how context (i.e., hospital of treatment) affects event rates [26].

In our study, to answer the above mentioned question, the existence of cluster effect (hospital effect) was tested by using a 2-level logistic modelling, where patients were nested into hospitals. The outcome variable was the PSI of interest, and the covariate variables were age, sex and the Elixhauser's comorbidities (EC) [28]. A model was tailored for each PSI (except MLM, which is considered a quasi-sentinel event), testing EC as covariates, taking into consideration the clinical reasoning -i.e., not all EC were used in all PSI-, and the magnitude of the association (OR ≥ 2) to avoid spurious findings due to the massive samples used in the study. The multilevel model was an extension of the previously estimated individual logistic models (c statistic was used to assess their goodness of fit) [30].

The degree of similarity of PSI events among providers was tested by using the rho statistic and its confidence intervals (type 1 error of 5%). The unobserved individual error followed a logistic distribution with individual variance equal to π^2^/3 [26].

Finally, the Median Odds Ratio (MOR) statistic (and its confidence intervals), a measure of the variation among clusters (hospitals in our study) was estimated by comparing pairs of patients with the same covariates from two, randomly chosen, different clusters [31]. MOR provides information on how heterogeneity across hospitals increases the individual odds of experiencing the outcome of interest.

#### Are measurements able to detect hospitals with a higher than expected number of cases?

This is a key question in the study as PSI are infrequent events, and imprecise measures and poor sensitivity are expected.

Given the existence of cluster effect, the natural way to assess the statistically significant difference between each hospital PSI rate and the expected rate, is to compute (and plot) shrunken residuals derived from the multilevel method. Shrunken residuals would disentangle the true hospital variation from that due to random [32]. ^.^

For the purposes of this study, the residual in each hospital and its standard error were estimated. The residual (μj) would represent the difference between the observed and the expected rate (μoj), being the expected the estimated average PSI rate for all the hospitals under study. Residual graphs exhibiting each hospital effect (and its confidence interval) around the average value (constant value for all hospitals as the expected one) were plotted. Residuals were assumed to follow a Gaussian distribution, N ~ (0, 1).

### Data sources

The 2005 and 2006 hospital discharges dataset (CMBD) was used to obtain numerators and denominators for each indicator -i.e. PSI inclusion and exclusion criteria. CMBD records the activity performed by all publicly funded hospitals across the country, enforced to provide this information in a yearly-basis. The register records, in a systematic and homogenous way, information from each patient discharge; specifically: age, sex, diagnosis of admission, secondary diagnoses (up to 30), length of stay, nature of the admission, discharge status and, diagnostic and therapeutic procedures performed. The register started off its activity in the mid 90s.

## Results

A total of 6.2 million discharges (between 171 and 175 hospitals depending on the indicator) were retrieved, once the new Spanish definitions were implemented. Admissions at risk ranged from 612,590 in post-operative sepsis to 2,954,018 in catheter-related infection. Adjusted-incidence ranged from 0.54 deaths per 1,000 patients admitted in low-mortality DRGs to 17.3 in postoperative sepsis per 1,000 eligible patients. (Table 1)

### Are differences among hospitals systematic?

**Figure 1:**
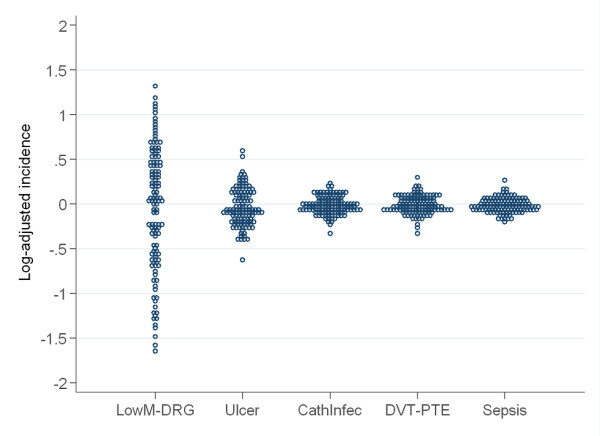
**Variation in adjusted-incidence by PSI**. Each dot represents the adjusted-incidence of adverse events in a specific hospital. Incidence is computed as a mean-centred log-incidence to allow the comparison among events with different basal incidence. Legend: y axis: log-adjusted-incidence. x axis: (left to right) Mortality in Low-Mortality DRGs, Decubitus Ulcer, Catheter-related Infection, Post-operative Pulmonary Embolism or Deep-vein Thrombosis and Post-operative Sepsis

The highest variation in the rate of adverse events among hospitals was observed in MLM [RV_5-95 _= 12.88 (CI95%: 9.36 to 14.98); RV_25-75 _= 7.06 (CI95%: 4.77 to 7.88)] being the smallest variation that in PS [RV_5-95 _= 1.31 (CI95%: 1.24 to 1.35); RV_25-75 _= 1.12 (CI95%:1.10 to 1.14)]. Figure 1 allows a visual comparison of the variation across the five PSI.

In accordance to the Empirical Bayes statistic, variation was observed to be systematic in all indicators, ranging from 0.19 (CI95%: 0.12 to 0.28) in the case of PE-DVT to 0.34 (CI95%: 0.25 to 0.45) in DU (Table 1).

### Do they measure differences among hospital-providers?

Multilevel logistic regressions were modelled to determine the effect of the hospital, once patient case-mix was adjusted. Although most of the variance was explained by patient-related factors ranging from 64% in PS to 79% in DU in accordance to the area under the curve, still a significant proportion of the variance was explained by the hospital: from a small rho value of 6% in the case of MLM (CI95%: 3% to 11%) to a high rho value of 24% (CI95%: 20% to 30%) in CRI. (Table 2)

**Table 2:** Multivariate analyses.


**Mortality in Low-mortality DRGs**	**Model 1** Hospital random effect	**Model 2 ** adds age-sex fixed-effect	**Model 3 ** adds comorbidity fixed-effect

***Patient variables (OR 95% CI)***			
Constant	0.001 (0.0005 to 0.007)		

***Measures of clustering***			
Hospital level variance (SE)	0.20 (0.07)		
Rho (95% CI)	0.06 (0.03 to 0.11)		
MOR (95% CI)	1.53 (1.35 to 1.81)		

**Decubitus Ulcer**	**Model 1** Hospital random effect	**Model 2** adds age-sex fixed-effect	**Model 3** adds comorbidity fixed-effect

***Patient variables (OR 95% CI)***			
Constant	0.008 (0.007 to 0.009)	0.00015 (0.00013 to 0.0002)	0.00018 (0.00015 to 0.0002)
Age		1.053 (1.052 to 1.055)	1.046 (1.045 to 1.047)
Sex		1.11 (1.08 to 1.14)	1.14 (1.09 to 1.17)
Paralysis			5.05 (4.81 to 5.31)
Other neurological disorders			3.74 (3.59 to 3.89)
Diabetes w chr. complications			1.87 (1.79 to 1.99)
Weight loss			5.16 (4.85 to 5.47)
Fluid And electrolyte disorders			2.97 (2.83 to 3.13

***Measures of clustering***			
Hospital level variance (SE)	0.54 (0.43 to 0.68)	0.46 (0.37 to 0.58)	0.38 (0.29 to 0.47)
Rho (95% CI)	0.14 (0.12 to 0.17)	0.12 (0.10 to 0.15)	0.10 (0.08 to 0.13)
MOR (95% CI)	2.01 (1.86 to 2.18)	1.91 (1.78 to 2.06)	1.79 (1.68 to 1.92)

**Catheter-related infection**	**Model 1** Hospital random effect	**Model 2** adds age-sex fixed-effect	**Model 3** adds comorbidity fixed-effect

***Patient variables (OR 95% CI)***			
Constant	0.001 (0.0009 to 0.0012)	0.0012 (0.001 to 0.0014)	0.0012 (0.001 to 0.0015)
Age		1.017 (1.016 to 1.018)	1.014 (1.013 to 1.015)
Sex		0.47 (0.44 to 0.49)	0.49 (0.46 to 0.51)
Peripheral vascular disease			2.05 (1.84 to 2.29)
Paralysis			2.20 (1.93 to 2.56)
Weight loss			3.63 (3.03 to 4.35)
Fluid And electrolyte disorders			2.36 (2.09 to 2.66)

***Measures of clustering***			
Hospital level variance (SE)	1.10 (0.84 to 1.44)	1.11 (0.85 to 1.45)	1.05 (0.80 to 1.38)
Rho (95% CI)	0.25 (0.20 to 0.31)	0.25 (0.20 to 0.31)	0.24 (0.20 to 0.30)
MOR (95% CI)	2.71 (2.39 to 3.13)	2.72 (2.39 to 3.14)	2.65 (2.38 to 3.06)

**Postoperative PE or DVT**	**Model 1** Hospital random effect	**Model 2** adds age-sex fixed-effect	**Model 3** adds comorbidity fixed-effect

***Patient variables (OR 95% CI)***			
Constant	0.0046 (0.0043 to 0.005)	0.00065 (0.00055 to 0.0007)	0.00059 (0.00052 to 0.0007)
Age		1.031 (1.029 to 1.032)	1.029 (1.028 to 1.03)
Sex		0.96 (0.92 to 1.002)	1.02 (0.82 to 1.06)
Pulmonary circulation disease			2.39 (2.16 to 2.66)
Paralysis			2.32 (2.07 to 2.58)
Lymphoma			2.14 (1.69 to 2.69)
Metastatic cancer			2.80 (2.56 to 3.03)
Solid tumor w/o metastasis			1.84 (1.65 to 2.03)
Coagulopthy			2.89 (2.56 to 3.25)
Weight loss			2.58 (2.25 to 2.94)

***Measures of clustering***			
Hospital level variance (SE)	0.26 (0.19 to 0.33)	0.24 (0.19 to 0.31)	0.20 (0.15 to 0.26)
Rho (95% CI)	0.07 (0.06 to 0.09)	0.07 (0.05 to 0.09)	0.06 (0.04 to 0.07)
MOR (95% CI)	1.62 (1.53 to 1.73)	1.59 (1.51 to 1.70)	1.53 (1.45 to 1.63)

**Postoperative sepsis**	**Model 1** Hospital random effect	**Model 2** adds age-sex fixed-effect	**Model 3** adds comorbidity fixed effect

*Patient variables (OR 95% CI)*			
Constant	0.014 (0.013 to 0.015)	0.006 (0.005 to 0.007)	0.0068 (0.0059 to 0.0077)
Age		1.022 (1.021 to 1.023)	1.019 (1.018 to 1.02)
Sex		0.63 (0.61 to 0.66)	0.64 (0.61 to 0.66)
Congestive heart failure			2.51 (2.36 to 2.69)
Paralysis			2.16 (1.95 to 2.39)
Weight loss			3.39 (2.91 to 3.89)

*Measures of clustering*			
Hospital level variance (SE)	0.28 (0.21 to 0.37)	0.31 (0.24 to 0.41)	0.30 (0.23 to 0.39)
Rho (95% CI)	0.08 (0.06 to 0.10)	0.09 (0.07 to 0.11)	0.08 (0.07 to 0.11)
MOR (95% CI)	1.65 (1.55 to 1.78)	1.70 (1.59 to 1.84)	1.69 (1.58 to 1.82)

Estimates for hospital (clustering) and individual effects

A Rho statistic value different to 0 represents the existence of cluster effect -the propensity of having an outcome is more similar among the patients within a hospital, that among patients from different hospitals; as for the magnitude of rho, the more the value, the greater the clustering

In the median case, as expressed by MOR, the variance among hospitals increased the individual risk expressed by ORs: by a 53% (MOR = 1.53 (CI95%:1.35 to 1.81) in the case of MLM, by a 79% in the risk of having DU attributable to the care received, by more than 2.6 times in the risk of experiencing a CRI, a 53% of suffering a PE-DVT after surgery and a 69% of having a PS.

### Are measurements precise enough and able to detect hospitals with a higher than expected number of cases?

**Figure 2:**
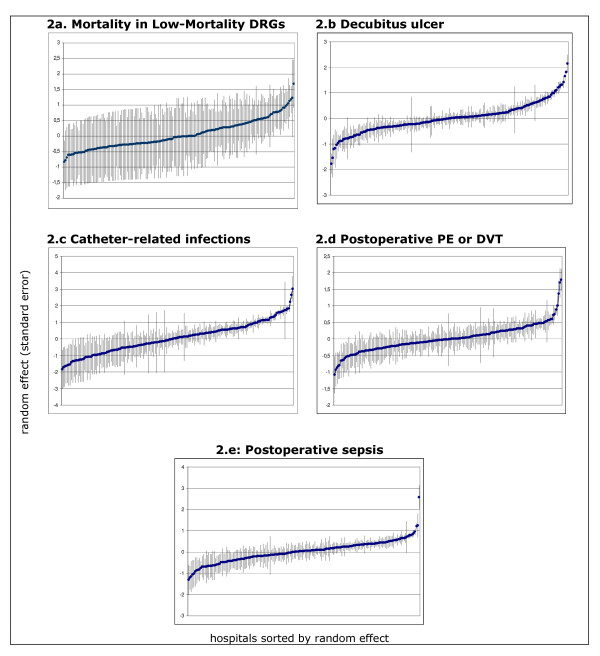
**Shrunken residuals (and standard errors) by PSI**. y axis: random effect (standard error). x axis: hospitals sorted by random effect **a**. **Mortality in Low-Mortality DRGs**. Note: Random effect (and standard error) after modelling the cluster effect. No patient variables were adjusted as Mortality in Low-Mortality DRGs is considered a sentinel-like event. **b. Decubitus ulcer**. Note: Random effect (and standard error) after modelling the cluster effect. Patient variables adjusted in the model were: age, sex, paralysis, other neurological disorders, diabetes with chronic complications, weight loss and fluid and electrolytic disorders. **c. Catheter-related infections**. Note: Random effect (and standard error) after modelling the cluster effect. Patient variables adjusted in the model were: age, sex, peripheral vascular disease, paralysis, weight loss, fluid and electrolytic disorders. **d**. **Postoperative PE or DVT**. Note: Random effect (and standard error) after modelling the cluster effect. Patient variables adjusted in the model were: age, sex, pulmonary circulation disease, paralysis, lymphoma, metastatic cancer, solid tumor w/o metastasis, coagulopathy and weight loss. **e**. **Postoperative sepsis**. Note: Random effect (and standard error) after modelling the cluster effect. Patient variables adjusted in the model were: age, sex, congestive heart failure, paralysis, and weight loss

As observed in Figure 2, after the risk adjustment, a remarkable amount of hospitals were found to be statistically positioned above the expected -average rate of adverse events predicted for the hospitals under study. So, 19 hospitals (11% of the sample) in the case of MLM, 46 hospitals (26%) in DU, 114 hospitals (35%) in CRI, 39 hospitals (22%) in PE-DVT, and 53 hospitals (31%) in PS were flagged as "underperformers".

## Discussion

Five PSI have been considered for empirical validation in public acute-care hospitals across Spain. All of them showed systematic variability (variation beyond chance), were proven to have cluster effect, and were able to detect hospitals above the expected. Nevertheless, several questions should be drawn out to provide a nuanced statement on their usefulness.

### Is the estimated variation systematic or due to chance?

Except in the case of MLM, since it is considered a quasi-sentinel event, we should know more about the basal distribution of adverse events to properly answer this question; however, we might assume, given the nature and rationale behind the safety indicators, that this distribution is expected to be close to zero.

Our approach was precisely based on testing the alternative hypothesis throughout the estimation of robust Empirical Bayes confidence intervals against zero as the null value. The precision of the estimated intervals together with the distance between the lower limit and the zero value (the closest figure corresponded to 0.12, in PE_DVT) support the hypothesis that the variation observed is systematic, rather than random.

### Is the observed variation due to hospital-providers, rather than to patients?

If this was not the case, PSI would not be useful in describing what they are aimed to, which is to elicit differences attributable to health care.

Our approach sought to elicit the hospital effect by estimating the existence of variation beyond the case-mix of patients treated -throughout the namely cluster effect. As mentioned in the results, in the studied PSI a noticeable part of variation was attributed to the hospital where the patients were treated. However, it might be argued that in a multilevel approach, this finding is quite dependant on the goodness of the risk adjustment -the worse the adjustment at patient level, the higher the proportion of variance that could be eventually explained by the hospital-level. This is particularly true in the case of studies using administrative data, where the limited information available on specific patient characteristics might reduce the goodness of risk-adjustment methods.

A way to mitigate this limitation is to reduce the extra-variance due to differences in case-mix that the model is unable to capture, by modelling the largest hospitals. These are teaching hospitals with more than 450 beds, able to provide high-tech services, and ultimately, homogeneous with regard to the patient case-mix, particularly in studies where sample size is as huge as ours.

The results of this exercise showed a significant reduction on rho-statistic values, backing the hypothesis that the strategy of risk-adjustment was missing some relevant patient characteristics. Even though this finding, cluster effect remained: rho-statistic equals 0.06 (CI95%: 0.03 to 0.11) in MLM; 0.05 (CI95%: 0.03 to 0.07) in DU; 0.10 (CI95%: 0.07 to 0.14) in CRI; 0.02 (CI95%: 0.01 to 0.03) in PE-DVT; and, 0.03 (CI95%: 0.03 to 0.05) in PS.

### Are results dependant on the coding practices affecting Elixhauser comorbidities?

A particular phenomenon that could also affect the cluster estimates, and ultimately the reliance on PSI, is the differential coding intensity across hospitals. In fact, the number of secondary diagnoses has been already proven to influence the international comparisons [21]. In theory, if this variation was closely related to coding intensity in hospitals, the cluster effect would suffer an important reduction when the number of secondary diagnoses was considered as a factor in the multilevel models; otherwise, it would be very much related to the patients, thus affecting the risk adjustment estimates.

For the purpose of this exploration the number of secondary diagnoses was categorized using the median value (4 secondary diagnoses) as a threshold. In general terms, when both models were compared, a clear reduction in the Elixhauser comorbidity β coefficients, together with stable rho-value estimates, were observed. (Additional file 1) Given that the number of secondary diagnoses absorbed part of the variance in the new model and beta coefficients changed, variation is also expected in the random effects estimation for each hospital. However, an excellent correlation (Pearson coefficient values) between the original random effects and the new ones was found: 0.83 in post-operative sepsis, 0.86 in post-operative PE-DVT, 0.94 in *decubitus *ulcer and 0.96 in Catheter-related infection. On the other hand, except in the case of decubitus ulcer the changes in the statistical nature of the random effect (i.e. hospitals found as statistically different that average turned into statistically similar, and the other way round) were null or negligible.

### Are PSI precise enough to detect hospitals with rates above the expected?

Although PSI are quite infrequent events, shrunken residuals from the multilevel analysis have been proven precise enough to detect hospitals above the expected. Figure 2 showed some quite straightforward images on this capacity. Nevertheless, determining in what manner cluster effect might be influenced by either outlier hospitals or the extra-variance attributable to the mix of hospitals within the sample is also needed.

With regard to the former, the estimation barely changed once those outlier values -easily identifiable at the two ends of the distribution in Figure 2- were excluded (data not shown). Most important is the latter one. To understand this effect, new residuals were estimated and plotted in those most *a priori *homogeneous centres, the largest ones as described in previous paragraphs. As observed, except in the case of MLM where heterogeneity across hospitals was the underlying reason for results (just 4 out of 47 hospitals were statistically above the expected in this second analysis), in the remaining PSI, this capacity held noticeably high: 23% of the hospitals were flagged above the expected in decubitus ulcer, up to 36% in catheter-related infection, 25% in the case of postoperative pulmonary embolism or deep vein thrombosis, and up to 28% in the case of postoperative sepsis. (Additional file 2)

### Should policy-makers and managers trust PSI?

Our work aimed at shedding light on some empirical properties that PSI are supposed to accomplish, in order to be useful for safety measurement and, ultimately, allow concerned users an informed quality management. Thus, representing systematic variation across providers -ruling out randomness as an alternative explanation of the differences-, and flagging hospitals as potential underperformers regardless the mix of patients they treat. However, a proper use requires debating upon two lessons learnt in this study, and reflecting upon other aspects that were not part of our work.

As for the lessons learnt with the studied PSI, due to the aforementioned flaws in adjusting patient-risks, we need to be aware that hospitals with more complexity might be signalled as false bad performers, particularly if they do not properly report secondary diagnoses. Secondly, the hospital effect (cluster effect) does exist, quite consistently throughout different statistic models; however, its magnitude clearly decreases when studying homogeneous hospital-providers. Although obvious, this message directly points towards comparing comparables, particularly, when risk adjustment is expected to be sub-optimal.

As for the reflection on other issues not addressed in this exercise, it is worth pointing out that the study of the empirical properties is just a partial view on PSI's validity. Further debate upon other validity issues ought to be pursued in order to fully trust on PSI usage. As for this purpose we have to be able to answer whether PSI measure what are supposed to measure. In this work, we have assumed construct validity since PSI were carefully developed for safety measurement purposes, [10,11] and face validity has been granted in advance for the Spanish case, by carrying out an *ad hoc *face-validity project [23]. However, criterion validity -the ability for an indicator to flag true positive cases and true negative cases by comparison with a gold standard- has to be specifically addressed, in context. Fortunately, for the Spanish NHS, a recent piece of research on surgical discharges shed some light on criterion validity [33]. In general terms, the five PSI were proven to have a quite good performance in terms of positive likelihood ratio (+LR). The most conservative estimation yielded a + LR of 26.8 in decubitus ulcer, a + LR of 406.3 in catheter-related infection, a + LR of 149.3 in PE-DVT and a + LR of 25.32 in postoperative sepsis. These figures seemed high enough to adopt the use of these PSI as a screening tool; except in the case of decubitus ulcer, clearly affected by underreporting (false negative cases) and the existence of present-on-admission ulcers (false positive cases).

Some additional effort should be made on evaluating the PSI stability over time (out of the scope of this work), but in the meantime, taking the studied PSI as screening tools, assessing wisely the limits pointed out along this work in specific contexts, might help to identify those centres from which best practice lessons can be drawn out and those where intervention is clearly needed.

## Conclusion

Five PSI showed reasonable empirical properties to screen healthcare performance in Spanish hospitals, particularly in the largest ones. However, ability to flag hospitals beyond the expected was limited in Mortality in Low-Mortality DRGs due to its larger standard errors, and risk for hospitals misclassification in decubitus ulcer remained.

## Abbreviations

ARQH: Agency for health research and quality; CI: Confidence interval; CMBD: Hospital discharges dataset; CRI: Infections due to medical care: including catheter-related infections; DRG: Diagnostic-related groups; DU: Decubitus ulcer; EB: Empirical Bayes Statistic; EC: Elixhauser's comorbidities; ICD 9^th^: International Classification of Diseases: version 9^th^; MLM: Death in low-mortality DRGs; MOR: Median Odds Ratio; NHS: Spanish National Health Service; OR: Odds ratio; PE-DVT: Postoperative pulmonary embolism or deep vein thrombosis; PS: Postoperative sepsis; PSI: Patient safety indicators; RV: Ratio of variation

## Competing interests

The authors declare that they have no competing interests.

## Authors' contributions

All the authors are guarantors of the study. All of them had full access to all the data, and take responsibility for the integrity and the accuracy of the analysis and results. EBD, SGA and SPM take responsibility on the article design, results interpretation and drafting. NML, BAT and JBP, contributed specifically to data management, and data analysis. All the authors read and approved the final manuscript.

## Pre-publication history

The pre-publication history for this paper can be accessed here:

http://www.biomedcentral.com/1471-2288/12/19/prepub

## Supplementary Material

Additional file 1 **The effect of the number of secondary diagnoses**. It shows the recalibration of each model using as a factor the number of secondary diagnoses. Tables show both the estimates before and after the adjustment. Click here for file

Additional file 2 **Shrunken residuals (and standard errors) by PSI**. It shows the residuals and standard errors for the largest hospitals in the sample (n = 47). Click here for file
