# Supplementary material for: Should policy-makers and managers trust PSI? An empirical validation study of five patient safety indicators in a national health service
Source: BMC Med Res Methodol. 2012 Feb 27;12:19. doi: 10.1186/1471-2288-12-19 (PMC3350386; doi:10.1186/1471-2288-12-19)
Supplement: Additional file 1 — The effect of the number of secondary diagnoses. It shows the recalibration of each model using as a factor the number of secondary diagnoses. Tables show both the estimates before and after the adjustment. [file 1471-2288-12-19-S1.DOC]

**Additional file 1**

**Estimation before and after adjusting the number of secondary diagnoses**

| **Decubitus Ulcer** | **before** | **after** |
| --- | --- | --- |
|
|  |  |  |
| ***Patient variables (β coeff., 95% CI)*** |  |  |
| Constant | -8.64 (-8.78 to -8.51) | -8.98 (-9.12 to -8.85 ) |
| Age | 0.045 (0.044 to 0.046) | 0.036 (0.035 to 0.038) |
| Sex | 0.13 (0.09 to 0.16) | 0.22 (0.19 to 0.25) |
| Paralysis | 1.62 (1.57 to 1.67) | 1.40 (1.35 to 1.44) |
| Other neurological disorders | 1.32 (1.28 to 1.36) | 1.18 (1.14 to 1.24) |
| Diabetes w chr. complications | 0.63 (0.58 to 0.69) | 0.34 (0.29 to 0.40) |
| Weight loss | 1.64 (1.58 to 1.70) | 1.42 (1.35 to 1.48) |
| Fluid And electrolyte disorders | 1.09 (1.04 to 1.14) | 0.83 (0.79 to 0.88) |
| Number of secondary diagnoses |  | 1.63 (1.59 to 1.68) |
| ***Measures of variation or clustering*** |  |  |
| Hospital level variance (SE) | 0.38 (0.29 to 0.47) | 0.35 (0.28 to 0.44) |
| Rho (95% CI) | 0.10 (0.08 to 0.13) | 0.10 (0.08 to 0.12) |
|  |  |  |
| **Catheter-related infection** | **before** | **After** |
|
|  |  |  |
| ***Patient variables (β coeff., 95% CI)*** |  |  |
| Constant | -6.73 (-6.94 to -6.52) | -7.74 (-7.92 to -7.55) |
| Age | 0.014 (0.013 to 0.015) | -0.005 (-0.007 to -0.004) |
| Sex | -0.72 (-0.78 to -.0.67) | -0.61 (-0.67 to -0.56) |
| Peripheral vascular disease | 0.72 (0.61 to 0.83) | 0.19 (0.08 to 0.30) |
| Paralysis | 0.79 (0.66 to 0.94) | 0.32 (0.18 to 0.46) |
| Weight loss | 1.29 (1.11 to 1.47) | 0.86 (0.68 to 1.03) |
| Fluid and electrolyte disorders | 0.86 (0.74 to 0.98) | 0.33 (0.21 to 0.44) |
| Number of secondary diagnoses |  | 2.62 (2.54 to 2.70) |
| ***Measures of variation or clustering*** |  |  |
| Hospital level variance (SE) | 1.05 (0.80 to 1.38) | 0.87 (0.66 to 1.15) |
| Rho (95% CI) | 0.24 (0.20 to 0.30) | 0.21 (0.17 to 0.26) |
|  |  |  |

| **Postoperative PE or DVT** | **before** | **After** |
| --- | --- | --- |
|
|  |  |  |
| ***Patient variables (β coeff., 95% CI)*** |  |  |
| Constant | -7.44 (-7.57 to -7.31) | -7.27 (-7.38 to -7.15) |
| Age | 0.029 (0.028 to 0.03) | 0.012 (0.010 to 0.013) |
| Sex | 0.02 (-0.02 to 0.06) | 0.19 (0.15 to 0.23) |
| Pulmonary circulation disease | 0.87 (0.77 to 0.98) | 0.26 (0.16 to 0.36) |
| Paralysis | 0.84 (0.73 to 0.95) | 0.34 (0.23 to 0.46) |
| Lymphoma | 0.76 (0.53 to 0.99) | 0.42 (0.18 to 0.65) |
| Metastatic cancer | 1.03 (0.94 to 1.11) | 0.64 (0.55 to 0.72) |
| Solid tumor w/o metastasis | 0.61 (0.50 to 0.71) | 0.34 (0.23 to 0.44) |
| Coagulopthy | 1.06 (0.94 to 1.18) | 0.51 (0.39 to 0.63) |
| Weight loss | 0.95 (0.81 to 1.08) | 0.47 (0.33 to 0.60) |
| Number of secondary diagnoses |  | 1.9 (1.85 to 1.96) |
| ***Measures of variation or clustering*** |  |  |
| Hospital level variance (SE) | 0.20 (0.15 to 0.26) | 0.19 (0.14 to 0.24) |
| Rho (95% CI) | 0.06 (0.04 to 0.07) | 0.05 (0.04 to 0.07) |
|  |  |  |
| **Postoperative sepsis** | **before** | **after** |
|
|  |  |  |
| ***Patient variables (β coeff., 95% CI)*** |  |  |
| Constant | -4.99 (-5.13 to -4.87) | -5.97 (-8.09 to -5.84) |
| Age | 0.019 (0.018 to 0.02) | -0.0005 (-0.0018 to 0.0007) |
| Sex | -0.45 (-0.49 to -0.41) | -0.16 (-0.19 to -0.12) |
| Congestive heart failure | 0.92 (0.85 to 0.99) | 0.28 (0.21 to 0.35) |
| Paralysis | 0.77 (0.67 to 0.87) | 0.19 (0.09 to 0.28) |
| Weight loss | 1.22 (1.07 to 1.36) | 0.61 (0.46 to 0.75) |
| Number of secondary diagnoses |  | 2.89 (2.82 to 2.95) |
| ***Measures of variation or clustering*** |  |  |
| Hospital level variance (SE) | 0.30 (0.23 to 0.39) | 0.30 (0.23 to 0.39) |
| Rho (95% CI) | 0.08 (0.07 to 0.11) | 0.08 (0.07 to 0.11) |
