# Supplementary material for: Should policy-makers and managers trust PSI? An empirical validation study of five patient safety indicators in a national health service
Source: BMC Med Res Methodol. 2012 Feb 27;12:19. doi: 10.1186/1471-2288-12-19 (PMC3350386; doi:10.1186/1471-2288-12-19)
Supplement: Additional file 2 — Shrunken residuals (and standard errors) by PSI. It shows the residuals and standard errors for the largest hospitals in the sample (n = 47). [file 1471-2288-12-19-S2.DOC]

**Additional file 2**

**Shrunken residuals (and standard errors) by PSI**

Largest Hospitals (n=47)

**Mortality in Low-Mortality DRGs**

* Random effect (and standard error) after modelling the cluster effect.No patient variables were adjusted as Mortality in Low-Mortality DRGs is considered a sentinel-like event.

**Decubitus ulcer**

* Random effect (and standard error) after modelling the cluster effect. Patient variables adjusted in the model were: age, sex, paralysis, other neurological disorders, diabetes with chronic complications, weight loss and fluid and electrolytic disorders.

**Catheter-related infections**

* Random effect (and standard error) after modelling the cluster effect. Patient variables adjusted in the model were: age, sex, peripheral vascular disease, paralysis, weight loss, fluid and electrolytic disorders.

**Postoperative PE or DVT**

* Random effect (and standard error) after modelling the cluster effect. Patient variables adjusted in the model were: age, sex, pulmonary circulation disease, paralysis, lymphoma, metastatic cancer, solid tumor w/o metastasis, coagulopathy and weight loss.

**Postoperative sepsis**

* Random effect (and standard error) after modelling the cluster effect. Patient variables adjusted in the model were: age, sex, congestive heart failure, paralysis, and weight loss.

Note: The y axis is representing the random effect for each hospital (and its confidence interval with z_α/2_=1.96); the x axis is representing hospitals sorted by random effect
